# A Novel YTHDF3-Based Model to Predict Prognosis and Therapeutic Response in Breast Cancer

**DOI:** 10.3389/fmolb.2022.874532

**Published:** 2022-06-09

**Authors:** Jie Liu, Jing Zhu, Xin Wang, Zhisheng Zhou, Haiyan Liu, Dajiang Zhu

**Affiliations:** ^1^ Department of Breast Cancer, Affiliated Foshan Maternity and Child Healthcare Hospital, Southern Medical University, Foshan, China; ^2^ Group of Ultrasonography in Obstetrics, Department of Obstetrics, Affiliated Foshan Maternity and Child Healthcare Hospital, Southern Medical University, Foshan, China

**Keywords:** breast cancer, YTHDF3, M6A, therapeutic response, model, nomogram, survival

## Abstract

**Background:** Due to high tumor heterogeneity, breast cancer (BC) patients still suffer poor survival outcomes. YTHDF3 plays a critical role in the prognosis of BC patients. Hence, we aimed to construct a YTHDF3-based model for the prediction of the overall survival (OS) and the sensitivity of therapeutic agents in BC patients.

**Methods:** Based on The Cancer Genome Atlas (TCGA, https://portal.gdc.cancer.gov/) database, we obtained BC patients’ data (*n* = 999) with YTHDF3 expression profiles. The association between YTHDF3 expression and 5-year OS was determined *via* Cox proportional hazards regression (CPHR) analysis. By integrating the variables, we established a prognostic nomogram. The model was estimated *via* discrimination, calibration ability, and decision curve analysis (DCA). The performance of the model was compared with the TNM stage system through receiver operating characteristic (ROC) curves and DCA. By means of the Genomics of Drug Sensitivity in Cancer (GDSC) database (https://www.cancerrxgene.org/), the therapeutic agents’ response was estimated. Gene set enrichment analysis (GSEA) demonstrated possible biological mechanisms related to YTHDF3. TIMER and CIBERSORTx were employed to analyze the association between YTHDF3 and tumor-infiltrating immune cells.

**Results:** The high YTHDF3 expression was significantly correlated with poor 5-year OS in BC patients. Through multivariate CPHR, four independent prognostic variables (age, TNM stage, YTHDF3 expression, and molecular subtype) were determined. On the basis of the four factors, a YTHDF3-based nomogram was built. The area under the curve (AUC) of the ROC curve for the model surpassed that of the TNM stage system (0.72 vs. 0.63, *p* = 0.00028). The model predictions showed close consistency with the actual observations via the calibration plot. Therapeutic response prediction was conducted in high- and low-risk groups and compared with each other. The BC patients with higher risk scores showed more therapeutic resistance than those with a lower risk score.

**Conclusion:** YTHDF3 was verified as a prognostic biomarker of BC, and a novel YTHDF3-based model was constructed to predict the 5-year OS of BC patients. Our model could be applied to effectively predict the therapeutic response of commonly used agents for BC patients.

## Introduction

Breast cancer (BC) has surpassed lung cancer and has become the most commonly diagnosed tumor in 2020 (Sung et al., 2021). In light of the estimation from the International Agency for Research on Cancer, the new cases of BC were about 2.3 million globally (Sung et al., 2021). BC is highly heterogeneous and has distinct biological features among molecular subtypes (Perou et al., 2000; Sorlie et al., 2001; Russnes et al., 2011; Metzger-Filho et al., 2012; Prabhu et al., 2017; Zhao and Rosen, 2021). Despite the great progress of therapeutic strategies, including immunotherapy, targeted therapy, chemotherapy, and endocrine therapy, the survival outcomes of many BC patients still remain poor (Harbeck et al., 2019; Barzaman et al., 2020). The tumor-node-metastasis (TNM) staging is widely adopted, but it is unable to precisely predict the prognosis of BC patients due to biological heterogeneity (Balachandran et al., 2015). Thus, it is vital to explore more new molecular biomarkers to help design better clinical treatment approaches and improve the predictive accuracy of the TNM staging system.

Several studies have suggested that the complicated signal pathways at genetic, transcriptomic, and epigenetic levels play important roles in BC oncogenesis and progression (Lehmann et al., 2011; Bianchini et al., 2016; Loibl and Gianni, 2017; Chen et al., 2020). N^6^-methyladenosine (m6A) is a noted posttranscriptional modification of coding RNAs and noncoding RNAs (ncRNAs) (Desrosiers et al., 1974; Wei et al., 1975; Coker et al., 2019; Huang et al., 2020). m6A can affect the metabolism of messenger RNAs (mRNAs), including splicing, export, translation, and decay (Pan et al., 2018), and plays broad roles in the functions and metabolism of various ncRNAs, such as long noncoding RNAs (lncRNAs), microRNAs, circular RNAs (circRNAs), small nuclear RNAs (snRNAs), and ribosomal RNAs (rRNAs) (Alarcon et al., 2015; Liu et al., 2015; Liu et al., 2017; Yang et al., 2017). Accumulating studies have shown that m6A is essential for BC cell proliferation, drug resistance, and antitumor immune response (Klinge et al., 2019; Li et al., 2019; Chen et al., 2020; He et al., 2021). m6A modification is a dynamically reversible biochemical process, including methylation and demethylation. Meanwhile, m6A-binding proteins (also called “readers”) are necessary for recognizing the chemical signatures (Wu et al., 2016). In light of the mechanism of m6A recognition, the readers can be classified into three categories: direct readers, indirect readers, and m6A switch readers. YTH domain-containing (YTHDC) proteins belong to the direct readers. There are five proteins forming the YTHDC protein family, namely, YTHDC1, YTHDC2, and YTHDF1-3. Recently, Chang et al. showed that YTHDF3 upregulation was essential for the process of metastasis, while stable ablation of YTHDF3 was capable to suppress brain metastasis (Chang et al., 2020). They uncovered the crucial role of YTHDF3 in BC brain metastasis.

Previous studies have demonstrated the effectiveness of immunotherapy in BC patients. Thus, the immune checkpoint inhibitors were applied in the clinic (Adams et al., 2019; Emens et al., 2019; Zhu et al., 2021). Nevertheless, the therapeutic resistance of chemotherapy, targeted therapy, and immunotherapy apparently decreased the survival time and resulted in poor prognosis in BC patients (Xu et al., 2017; Lainetti et al., 2020; Hanna and Balko, 2021).

Herein, our study aimed to explore the YTHDF3 expression with the prognosis of BC patients, as well as the immune infiltrates. Furthermore, we attempted to establish a YTHDF3-based nomogram to assess the individual risk of 5-year OS in BC patients. Based on the clinical tool, we sought to predict the therapeutic responses of commonly used drugs in BC patients.

## Materials and Methods

### Patients and Study Design

Level 3 RNA sequencing profiles of BC patients and accompanying clinical characteristic data were obtained from TCGA database. We excluded the samples which had absent or deficient information on age, TNM stage, overall survival (OS) time, or survival status. The inclusion criteria were as follows: 1) local invasive BC identified by using the pathological method; 2) OS time was longer than 1 month; and 3) both survival data and RNA expression profiles were completely available. Clinical features contained TNM stage, T stage, N stage, age, estrogen receptor (ER), progesterone receptor (PR), HER-2 status, tumor subtypes, OS, and OS time. Thus, 999 BC patients were divided into two groups (the high- and the low-expression groups) depending on the median value of YTHDF3 expression. The correlation between YTHDF3 expression and the clinicopathological factors was determined. Meanwhile, we analyzed the YTHDF3 differential expression in 27 diverse kinds of cancers.

### Immune Infiltrate Analysis

The Tumor Immune Estimation Resource (TIMER) database provided a comprehensive analysis of immune infiltrates of various types of tumors (https://cistrome.shinyapps.io/timer). We performed the YTHDF3 expression in BC with the abundance of tumor-infiltrating immune cells (TIICs) through gene modules in TIMER. Furthermore, by using CIBERSORTx (http://cibersortx. Stanford. edu/), which is a deconvolution algorithm dependent on gene expression, we analyzed the immune microenvironment to explore the correlation between TIICs and YTHDF3 expression in BC patients.

### Gene Set Enrichment Analysis

In our report, 999 BC cases were divided into two groups (the high- and low-expression groups) according to the median value of YTHDF3 expression. We performed GSEA to determine the biological process that is enriched by YTHDF3 in BC patients. We selected “c2.cp.kegg.v6.2.symbols.gmt” as the gene set. The permutation number was set at 1,000 times, the minimum and maximum number sizes for gene analysis at 15 and 1,000, respectively. Gene sets with a nominal *p*-value < 0.05 or a false discovery rate (FDR) q < 0.25 were the significant threshold.

### Construction and Evaluation of the YTHDF3-Based Nomogram

According to the analysis of univariate and multivariate CPHR, the YTHDF3-based model was conducted. The performance of the nomogram was quantified in reference to the ability of discrimination, calibration, and clinical utility. The score test was applied to estimate the goodness of fit of the model. The AUC of ROC curves was utilized to figure out the discrimination ability. The calibration ability was assessed *via* a calibration curve. In addition, DCA was used to illustrate the clinical utility of the model.

### Therapeutic Response Prediction

We employed “pRRophetic” R package to estimate the chemotherapy and the targeted therapy response combined with the Genomics of Drug Sensitivity in Cancer (GDSC, https://www.cancerrxgene.org) (Geeleher et al., 2014). Five commonly used chemotherapeutic agents vinorelbine (Adamo et al., 2019; Falvo et al., 2021; Wang et al., 2021), cisplatin (Hu et al., 2015; Jovanović et al., 2017; Tung et al., 2020), methotrexate (Cameron et al., 2017; Lu et al., 2020; Shakeran et al., 2021), doxorubicin (Mackey et al., 2016; Ganz et al., 2017; Schneeweiss et al., 2022), paclitaxel(Diéras et al., 2020; Fernandez-Martinez et al., 2020; Tolaney et al., 2021), and small-molecule inhibitor targeting EGFR lapatinib (Cortés et al., 2015; Powles et al., 2018; Johnston et al., 2021) were selected and kept the default values for all parameters. The response prediction relied on IC50 (the half-maximal inhibitory concentration) of each sample. The method for estimating the value of IC50 is ridge regression analysis. Through 10× cross-validation, the prediction accuracy was assessed.

### Statistical Analysis

The Wilcoxon rank-sum test was applied to compare the expression of YTHDF3 in BC and normal groups. We used the Kaplan–Meier (K-M) method and log-rank test to analyze the OS of two groups (the high- and low-YTHDF3 expression groups), respectively. CPHR analysis was conducted to screen the independent factors of OS. Ridge regression analysis was performed to estimate the value of IC 50. The association between YTHDF3 and TILs was determined *via* the TIMER and CIBERSORTx algorithm. R software (version 4.0.3) was applied to determine the correlation between the expression of the RNA-seq gene and clinical parameters in BC. A *p* value <0.05 meant a significant difference.

## Results

### Baseline Characteristics of Patients

The clinical characteristics of 999 BC patients, including age, TNM stage, molecular subtypes, ER, PR, HER-2, and vital status, were extracted from TCGA database. In light of the inclusion and exclusion criteria, 999 patients were involved in later analysis. In our research, the patients’ age when diagnosed ranged from 26–89 years, and the medium of age was 57–91 years. The median follow-up time was 42.4 months, with median ranged from 1 to 286.8 months. The result was shown in Table 1.

**TABLE 1:** Patient demographics and clinical characteristics.

Characteristic	No. of patients (%)
Total number	999 (100)
Age (years)	58 (48, 67)
T stage	
1	271 (27.1)
2	567 (56.8)
3	124 (12.4)
Unknown	37 (3.7)
**N stage**	
0	461 (46.2)
1	343 (34.3)
2	108 (10.8)
3	70 (7.0)
Unknown	17 (1.7)
**TNM stage**	
I	167 (16.7)
II	575 (57.6)
III	240 (24.0)
Unknown	17 (1.7)
Tumor type	
HR+/HER-2-	559 (56.0)
HR+/HER-2+	138 (13.8)
HR-/HER-2+	36 (3.6)
TNBC	137 (13.7)
Unknown	129 (12.9)
ER status	
Positive	208 (20.8)
Negative	751 (75.2)
Unknown	40 (4.0)
**PR status**	
Positive	299 (29.9)
Negative	659 (66.0)
Unknown	41 (4.1)
HER2 status	
Positive	697 (69.8)
Negative	174 (17.4)
Unknown	128 (12.8)

### High YTHDF3 Expression Serves as an Indicator for Poor Prognosis in BC

**FIGURE 1:**
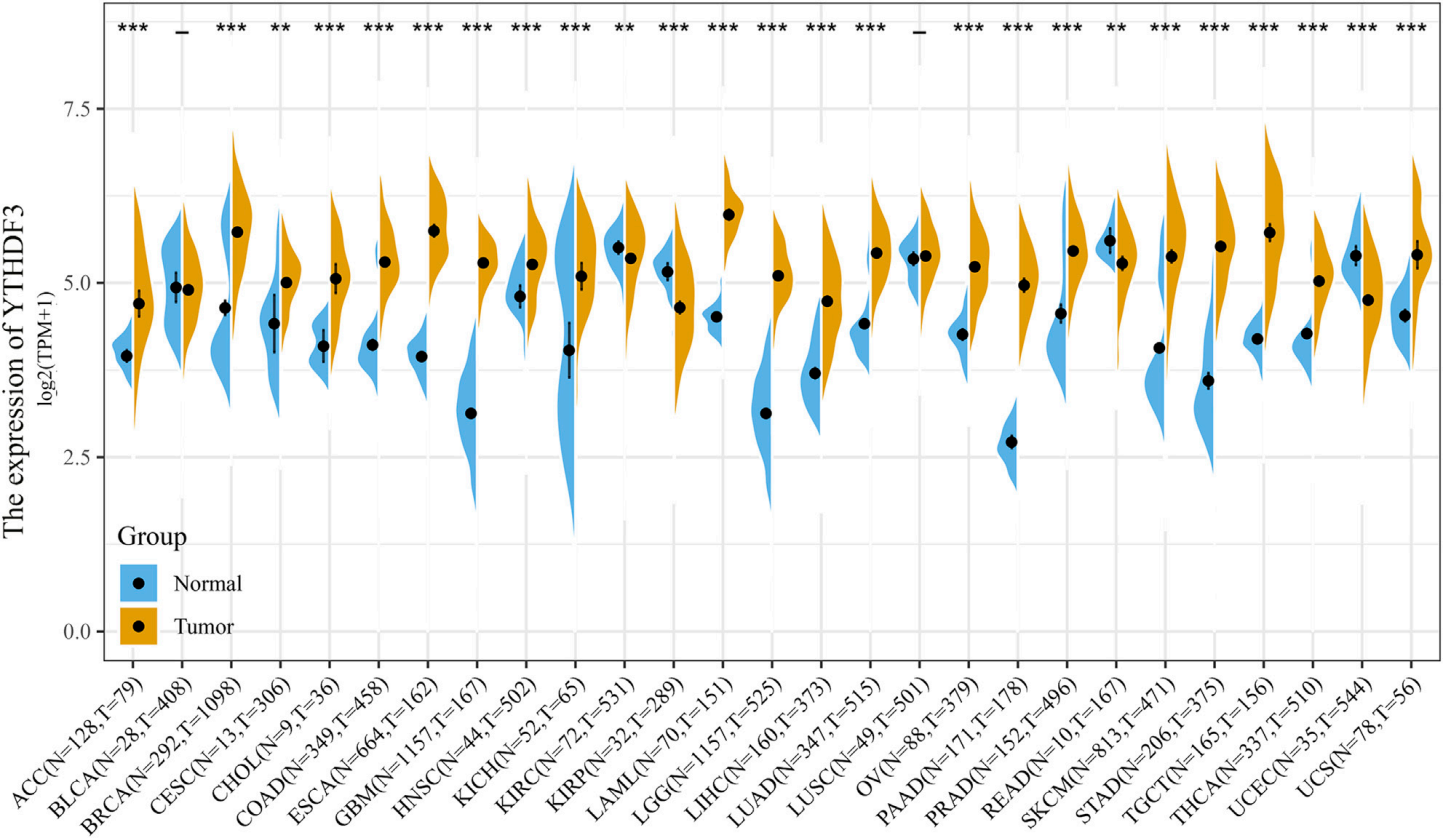
YTHDF3 differential expression between normal and cancer tissues in 27 kinds of cancer was analyzed by TCGA and GTEx database. - no statistical difference, **p < 0.01. ***p < 0.001.

In TCGA database, the YTHDF3 differential expression was analyzed between tumor and normal tissues in 27 diverse cancer types (Figure 1). The YTHDF3 expression was significantly highly upregulated in 1,098 BC patients versus 292 normal group individuals (*p* < 0.001), as well as in 21 of the 27 analyzed cancer types (Figure 1).

**FIGURE 2:**
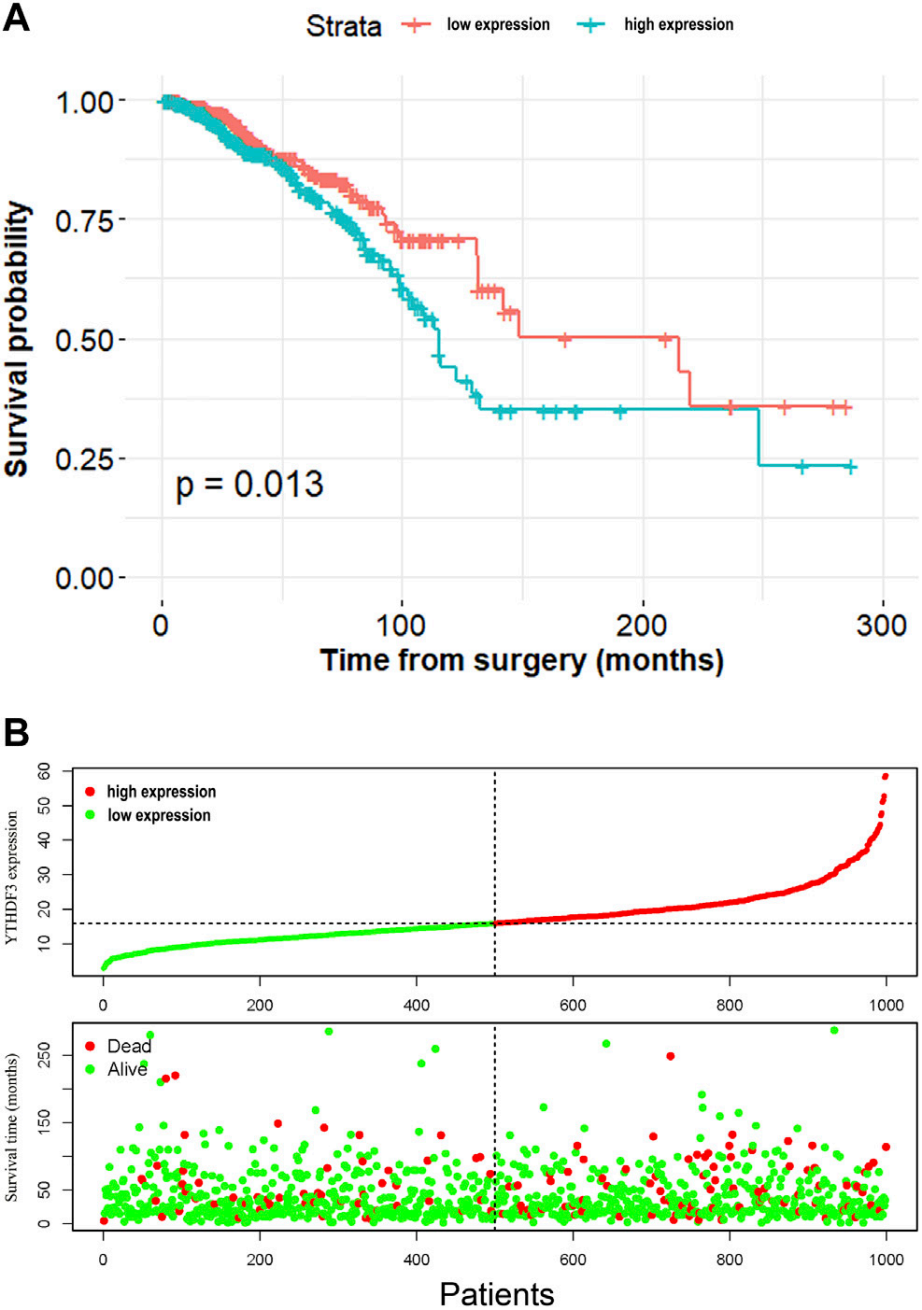
**(A)** K-M curves of patients in high- and low-expression groups based on the median value of YTHDF3 expression. **(B)** YTHDF3 expression distribution and survival status.

We conducted the CPHR analysis to figure out the correlation between OS and the YTHDF3 expression, as well as other clinical characteristics in BC patients. As Table 2 shows, univariate analysis suggested that several factors, including YTHDF3 (HR = 1.02, *p* = 0.003), age (HR = 1.032, *p* < 0.001), and TNM stage III are significantly associated with OS. Multivariate CPHR uncovered that YTHDF3 expression was an independent prognostic factor for OS. The expression distribution and profiles of YTHDF3 and survival status of BC patients are demonstrated in Figure 2A.

**TABLE 2:** Univariate and multivariate Cox proportional hazards regression analyses in BC patients.

Variable	Univariate analysis		Multivariate analysis	
	HR (95% CI)	*p* value	HR (95% CI)	*p* value
Age	1.032(1.018–1.047)	<0.001	1.033(1.019–1.048)	<0.001
TNM stage				
I	Referent	—	Referent	—
II	1.547 (0.881–2.716)	0.129	1.609 (0.912–2.839)	0.101
III	3.096 (1.733–5.533)	<0.001	3.375 (1.875–6.073)	<0.001
Unknown	7.337 (3.203–16.809)	<0.001	6.718 (2.835–15.918)	<0.001
Tumor subtypes				
HR+/HER2-	Referent	—	Referent	—
HR+/HER2+	1.472 (0.837–2.589)	0.179	1.53 (0.864–2.707)	0.145
HR-/HER2+	2.225 (0.952–5.201)	0.065	1.49 (0.619–3.582)	0.374
TNBC	1.588 (0.954–2.645)	0.075	1.878 (1.123–3.142)	0.016
Unknown	1.671 (1.074–2.6)	0.023	1.341 (0.859–2.094)	0.197
ER status				
Negative	Referent	—	—	—
Positive	0.767 (0.522–1.125)	0.175	—	—
Unknown	1.781 (0.788–4.024)	0.165	—	—
PR status				
Negative	Referent		—	—
Positive	0.813 (0.568–1.163)	0.258	—	—
Unknown	1.842 (0.828–4.099)	0.134	—	—
HER2 status				
Negative	Referent		—	—
Positive	1.447 (0.895–2.338)	0.131	—	—
Unknown	1.502 (0.992–2.275)	0.055	—	—
YTHDF3	1.028 (1.009–1.047)	0.003	1.02 (1–1.04)	0.047

In order to determine the prognostic role of YTHDF3 in BC, the expression data were separated into high- and low-risk groups in light of the median value of YTHDF3 expression. The K-M method was conducted to describe the OS of two groups. The log-rank test of OS curves showed that elevated YTHDF3 expression was combined with shorter OS time in BC patients (*p* = 0.013).

### YTHDF3 Expression Is Significantly Related to Immune Infiltration

**FIGURE 3:**
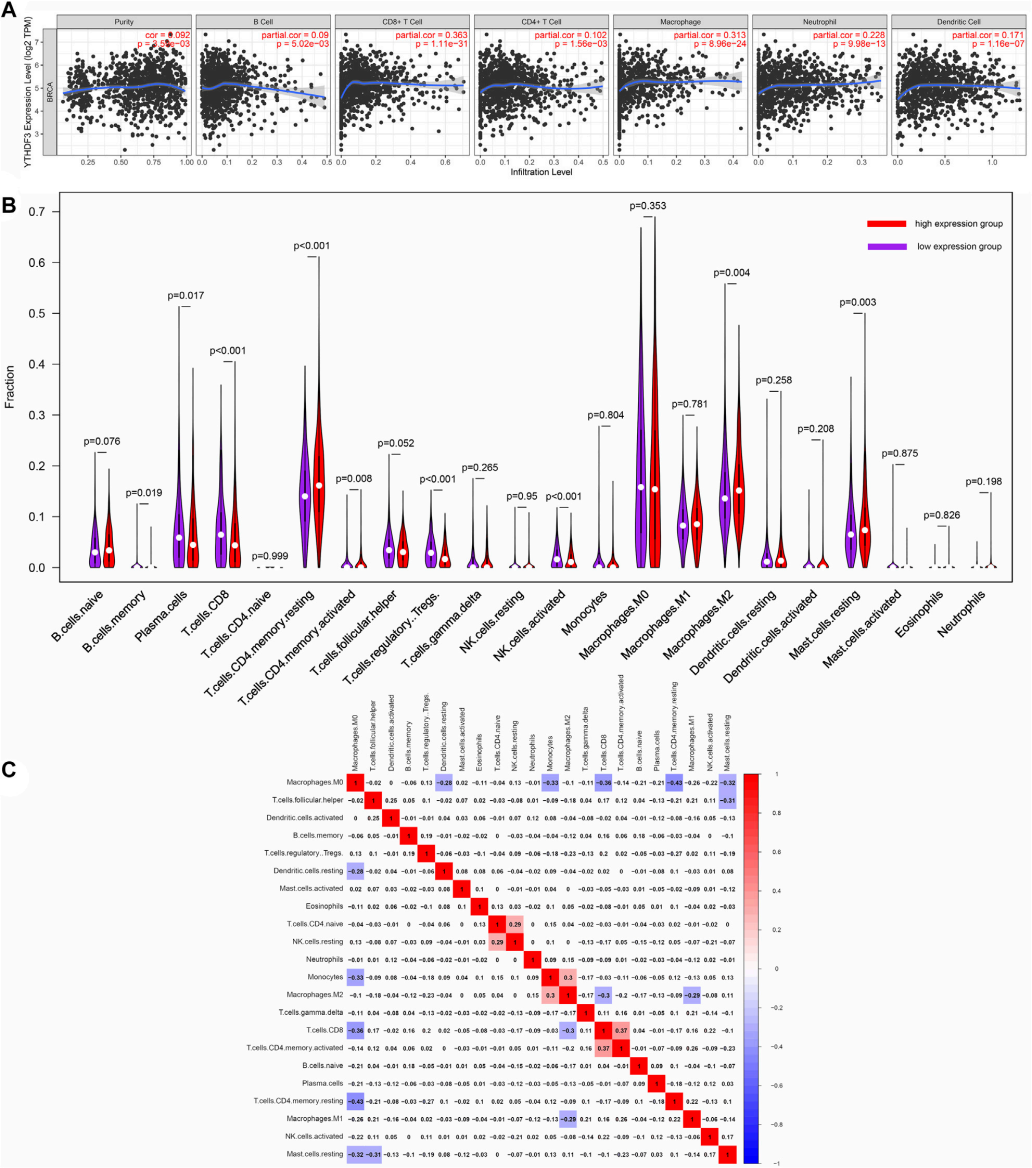
**(A)** Relations between YTHDF3 expression and the infiltration of immune cells. **(B)** Diverse fractions of immune cell subpopulations in high- and low-YTHDF3 expression groups. **(C)** Heatmap of immune cells in BC.

The tumor-infiltrating lymphocytes (TILs) can independently predict the survival status (Azimi et al., 2012). Furthermore, TILs play an essential role in facilitating a favorable fate in BC patients (Stanton and Disis, 2016). Hence, we explored the association between immune infiltration and YTHDF3 expression in BC *via* TIMER. As shown in Figure 3A, YTHDF3 expression positively related to the levels of B cells (*p* = 5.02 × 10^–3^), CD4^+^ T cells (*p* = 1.56 × 10^–3^), CD8^+^ T cells (*p* = 1.11 × 10^–31^), macrophages (*p* = 8.96 × 10^–24^), neutrophils (*p* = 9.98 × 10^–13^), and dendritic cells (*p* = 1.16 × 10^–7^). These results revealed that YTHDF3 was a crucial part in the tumor immune infiltration of BC.

Meanwhile, we attempted to determine if the immune infiltrating microenvironment varied according to the high and low YTHDF3 expression levels. The CIBERSORTx algorithm (https://cibersortx.stanford.edu/) was utilized to identify different infiltrating levels of diverse immune cell subtypes in the high- and low-YTHDF3 expression groups (Figure 3B). Among the 22 subpopulations of immune cells, plasma cells, CD8^+^ T cells, memory B cells, resting memory CD4^+^ T cells, regulatory T cells, activated memory CD4^+^ T cells, activated NK cells, resting mast cells, and M2 macrophages were considerably influenced by YTHDF3 expression. Specifically, memory B cells (*p* = 0.019), plasma cells (*p* = 0.017), activated NK cells (*p* < 0.001), regulatory T cells (*p* < 0.001), and M2 macrophages (*p* = 0.004) apparently increased in low-YTHDF3 expression group. In contrast, CD8^+^ T cells (*p* < 0.001), resting memory CD4^+^ T cells (*p* < 0.001), resting mast cells (*p* = 0.003), and activated memory CD4^+^ T cells (*p* = 0.008) were significantly elevated in the high-YTHDF3 expression group.

The possible link among 22 subtypes of immune cells was also evaluated (Figure 3C). The heatmap demonstrated that the ratios of diverse TILs ranged from weakly to moderately correlated.

### Gene Set Enrichment Analysis in High- and Low-YTHDF3 Expression Groups

**FIGURE 4:**
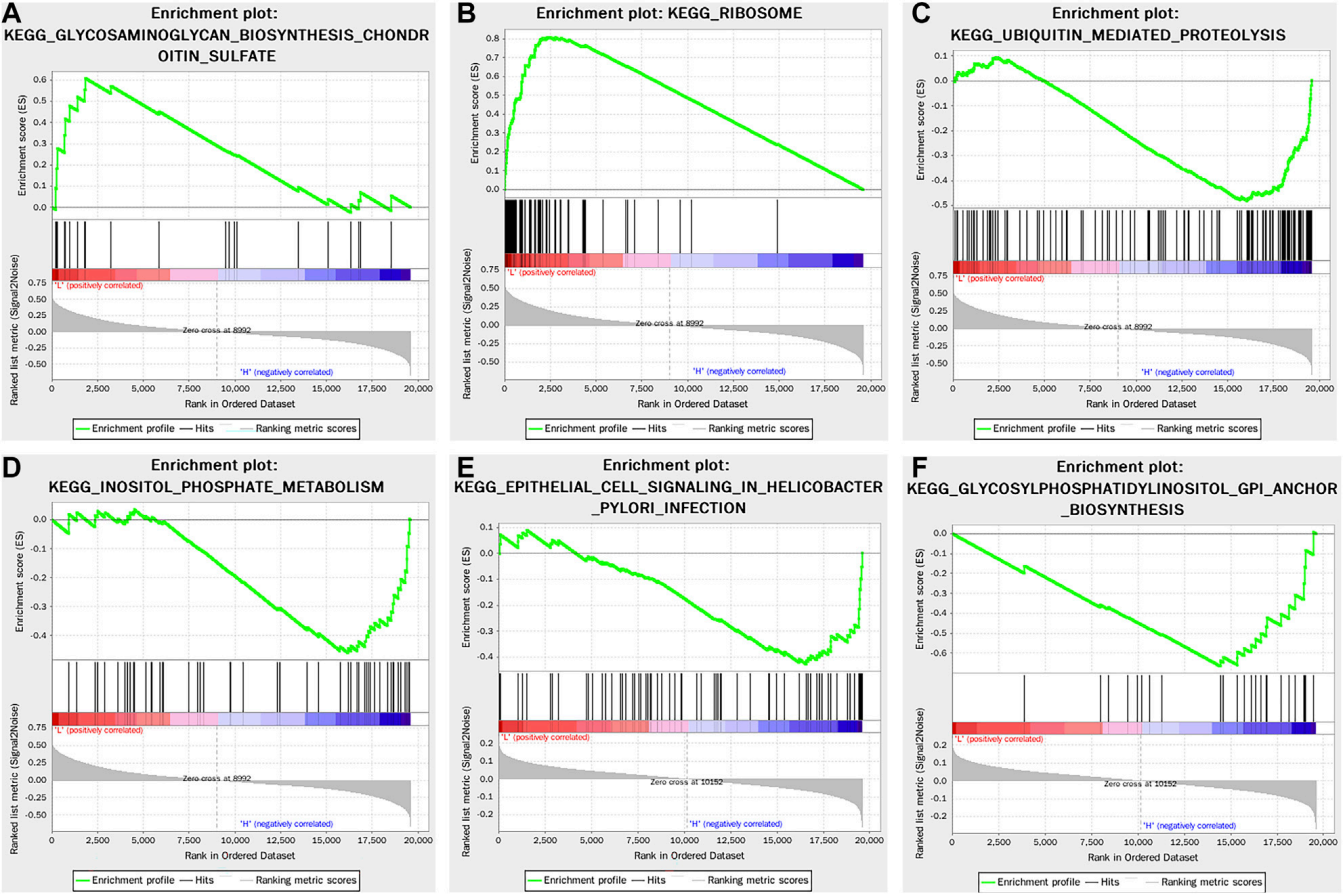
Enrichment plots from GSEA using the single-gene method of YTHDF3. The results showed the four most positively related pathways and two negatively related pathway. **(A)** Enrichment plot: KEGG_GLYCOSAMINOGLYCAN_BIOSYNTHESIS_CHONDROITIN_SULFATE **(B)** Enrichment plot: KEGG_RIBOSOME **(C)** Enrichment plot: KEGG_UBIQUITIN_MEDIATED_ PROTEOLYSIS **(D)** Enrichment plot: KEGG_INOSITOL_PHOSPHATE_METABOLISM **(E)** Enrichment plot: KEGG_EPITHELIAL_CELL_SIGNALING_IN_HELICOBACTER_PYLORI_INFECTION **(F)** Enrichment plot: KEGG_GLYCOSYLPHOSPHATIDYLINOSITOL_GPI_ANCHOR_BIOSYNTHESIS.

GSEA was performed to select the YTHDF3-related signaling pathway in BC. In light of the median YTHDF3 expression value, TCGA data were divided into the high- and low-YTHDF3 expression groups. A nominal *p*-value <0.05 and an FDR <0.25 were regarded as significant. The results of GSEA demonstrated that five KEGG items (selected based on the NES) were significantly differentially enriched in the high-YTHDF3 expression group, including ubiquitin-mediated proteolysis, inositol phosphate metabolism, epithelial cell signaling in *Helicobacter pylori* infection, and glycosylphosphatidylinositol GPI anchor biosynthesis, whereas the gene set involved in the low-YTHDF3 expression group was glycosaminoglycan biosynthesis chondroitin sulfate and ribosome. All snapshots of enrichment results are shown in Figure 4.

### The Performance of YTHDF3-Based Model

**FIGURE 5:**
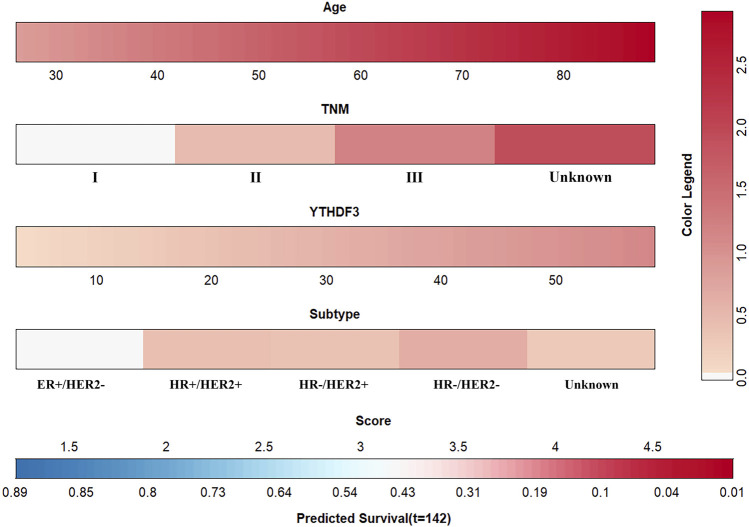
Prognostic model for predicting 5-year OS in BC patients.

**FIGURE 6:**
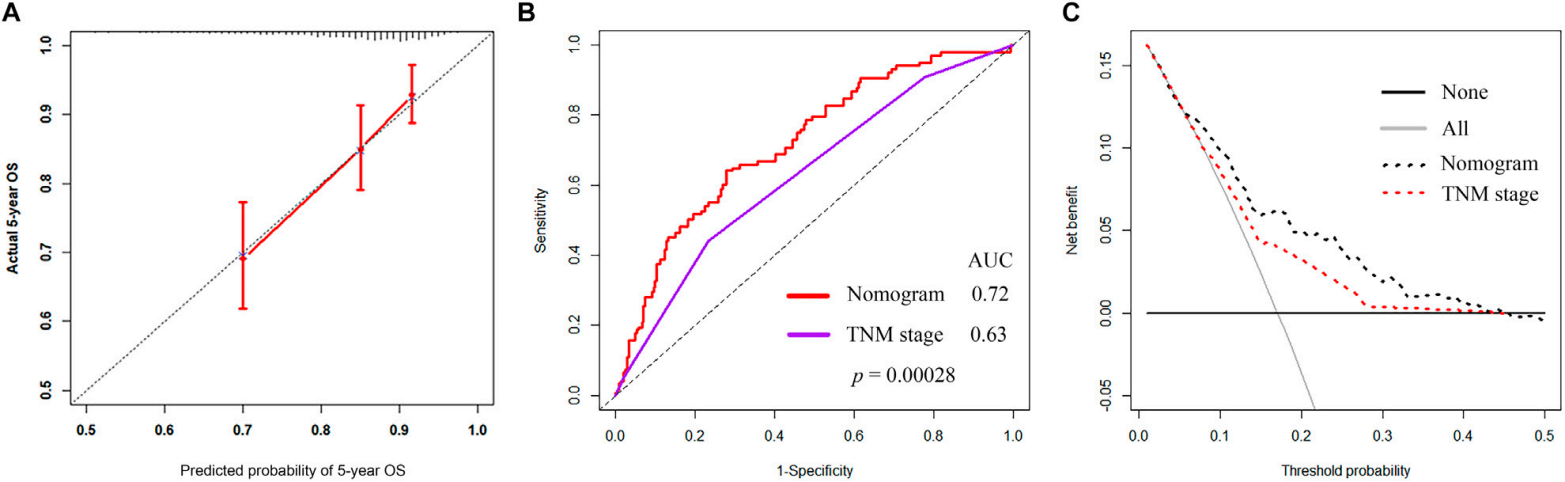
**(A)** Calibration curve of the model to predict 5-year OS. **(B)** Time-dependent ROC curves of the model and the TNM stage. **(C)** DCA of the nomogram and TNM stage for predicting 5-year OS in BC patients.

Age, TNM stage, BC subtype, and the YTHDF3 expression value were determined as independent variables through the multivariate CPHR analysis (Table 2). The YTHDF3-based nomogram is shown in Figure 5A. The result of the score test was 77.43, and the *p* value was 5e-13, which indicated good model fitness. The AUC of the model was 0.721 (95% CI: 0.654–0.789), which suggested good recognition ability of the model and prediction superiority over the TNM staging system (AUC: 0.72 vs. 0.3, *p* < 0.00028) (Figure 6B). The calibration curve showed high consistency between the predicted 5-year OS rate by model and the actual observed results (Figure 6A). Furthermore, with respect to clinical utility, the YTHDF3-based model was better than the TNM staging system on account of the wider range of threshold probability *via* DCA (Figure 6C).

### The role of the YTHDF3-Based Nomogram in Predicting Sensitivity to Therapeutic Agents in BC Patients

**FIGURE 7:**
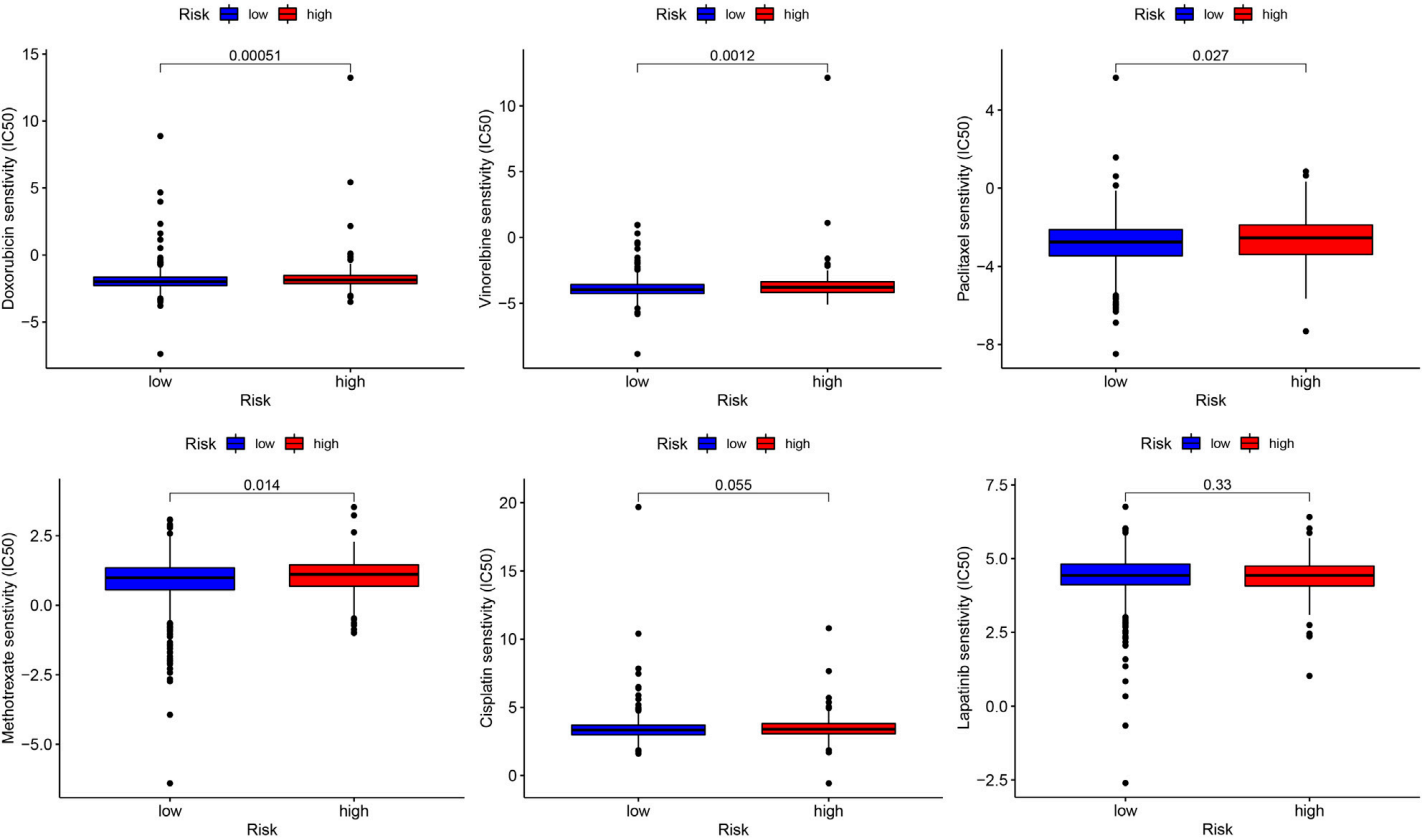
Role of the YTHDF3-based nomogram in predicting the sensitivity to therapeutic drugs. The box plots display the estimated IC50 of vinorelbine, cisplatin, doxorubicin, methotrexate, paclitaxel, and lapatinib.

Six commonly used BC therapeutic drugs (doxorubicin, vinorelbine, paclitaxel, methotrexate, cisplatin, and lapatinib) were selected as the candidates, which might show drug resistance in the high-risk group *via* the estimation of the IC_50_ values. As displayed in Figure 7, the estimated IC50 values of doxorubicin, vinorelbine, paclitaxel, and methotrexate significantly increased in the high-risk group. There were no estimated IC50 value differences of cisplatin and lapatinib observed between the high- and low-risk groups.

## Discussion

Our study demonstrated that higher YTHDF3 expression meant worse OS and was associated with lower TIL abundance. Based on the results of multi-CPHR, we built a YTHDF3-based nomogram combined with other clinical features. The model demonstrated superior prognostic accuracy and better clinical values than the TNM stage. Finally, we performed therapeutic response prediction of BC patients in the high- and low-risk groups. The BC patients with high-risk scores were predicted to be less sensitive to several commonly used therapeutic agents, including doxorubicin, paclitaxel, methotrexate, and vinorelbine.

Several studies had proposed nomograms to predict OS in BC patients. Zhao et al. reported that the models incorporating age, tumor stage, and molecular subtype could predict the OS in BC patients, respectively. However, all those models omitted the predictive value of genes associated with m6A modification. As mentioned in prior research studies, YTHDF3 efficiently improved the translational process of its targets and depended on m6A methylation (Coots et al., 2017; Li et al., 2017; Shi et al., 2017). YTHDF3 overexpression contributed to BC progression (Anita et al., 2020) and facilitates the BC brain metastasis progress (Chang et al., 2020). BC is a heterogeneous tumor with diverse prognoses and different drug resistances, and even the patients have the identical TNM stage. Here, we managed to establish and calibrate a YTHDF3-based nomogram for effectively promoting the 5-year OS predictive accuracy of the TNM stage in BC patients.

In order to predict the drug sensitivity, several commonly used cytotoxic drugs and the small-molecule inhibitor lapatinib were screened out. The estimated IC50 values of these drugs were remarkably increased in the high-risk group. The higher IC50 values meant that the BC patients demonstrated less susceptibility to the agents they have taken. Anthracyclines and paclitaxel play fundamental roles in BC chemotherapy. Nevertheless, due to the high rate of chemoresistance, the survival outcome of BC patients is unsatisfactory. If oncologists were available to recognize the worthless drugs before making a prescription using a tool, the heavy medical burden on patients and society may be reduced. Fortunately, this study proposed a novel nomogram based on YTHDF3 expression, which was valuable in guiding the selection of therapeutic agents. Furthermore, it had the capability of picking out those who might be the potential ineffective candidates.

Equally important, we employed the TIMER database to reveal connections between immune infiltration levels and YTHDF3 expression in BC. The associations of YTHDF3 expression with CD8+ T cells, dendritic cells, neutrophils, and macrophages were the strongest. Meanwhile, NK cells, macrophages, and mast cells showed a moderate to strong positive linkage with YTHDF3 expression. According to the CIBERSORTx result, the infiltration levels of plasma cells, Treg cells, activated NK cells, and M2 macrophages were decreased in the high-YTHDF3 expression group compared with the low-expression one. NK cells are cytotoxic and secrete cytokines and cytotoxic granules, which could suppress the cancer cell proliferation and mediate the antitumor process of NK cells (Müller-Hermelink et al., 2008; Vivier et al., 2011; Cerwenka and Lanier, 2016). Muntasell et al. showed that NK cell infiltration is associated with anti-HER-2 treatment in HER-2-positive BC patients (Muntasell et al., 2019). Overexpression of YTHDF3 might inhibit the function and efficiency of NK cells in antitumor immune response, which could be one of the routes that YTHDF3 affects the tumor immune. The closed correlation implies a likely mechanism by which YTHDF3 regulates the biological functions of tumor-infiltrating immune cells in BC patients.

Several limitations can be mentioned in our research. First, the role YTHDF3 played in therapeutic agents’ resistance and the potential molecular mechanism should be further investigated using additional experiments. Second, a different population from another center or dataset is needed to externally validate the prediction nomogram. Finally, we could not assess the influence of postoperative information (chemoradiotherapy and endocrine therapy) due to the unavailability of data from TCGA database.

## Conclusion

In sum, we confirmed the significant association between YTHDF3 expression and the 5-year OS in BC patients. Then, a novel nomogram was efficiently conducted to predict 5-year OS by integrating YTHDF3 expression, age, molecular subtype, and TNM stage. Our model can be utilized to predict the sensitivity of therapeutic agents and guide oncologists to select the appropriate therapeutic regimens.

## Data Availability

The original contributions presented in the study are included in the article/Supplementary Material, further inquiries can be directed to the corresponding authors.

## References

[B1] Adams S. Loi S. Toppmeyer D. Cescon D. W. De Laurentiis M. Nanda R. (2019). Pembrolizumab Monotherapy for Previously Untreated, PD-L1-Positive, Metastatic Triple-Negative Breast Cancer: Cohort B of the Phase II KEYNOTE-086 Study. Ann. Oncol. 30 (3), 405–411. 10.1093/annonc/mdy518 30475947

[B2] Alarcón C. R. Lee H. Goodarzi H. Halberg N. Tavazoie S. F. (2015). N6-methyladenosine marks Primary microRNAs for Processing. Nature 519 (7544), 482–485. 10.1038/nature14281 25799998 PMC4475635

[B3] Anita R. Paramasivam A. Priyadharsini J. V. Chitra S. (2020). The m6A Readers YTHDF1 and YTHDF3 Aberrations Associated with Metastasis and Predict Poor Prognosis in Breast Cancer Patients. Am. J. Cancer Res. 10 (8), 2546 32905518 PMC7471347

[B4] Azimi F. Scolyer R. A. Rumcheva P. Moncrieff M. Murali R. McCarthy S. W. (2012). Tumor-infiltrating Lymphocyte Grade Is an Independent Predictor of sentinel Lymph Node Status and Survival in Patients with Cutaneous Melanoma. Jco 30 (21), 2678–2683. 10.1200/JCO.2011.37.8539 22711850

[B5] Balachandran V. P. Gonen M. Smith J. J. DeMatteo R. P. (2015). Nomograms in Oncology: More Than Meets the Eye. Lancet Oncol. 16 (4), e173–e180. 10.1016/s1470-2045(14)71116-7 25846097 PMC4465353

[B6] Barzaman K. Karami J. Zarei Z. Hosseinzadeh A. Kazemi M. H. Moradi-Kalbolandi S. (2020). Breast Cancer: Biology, Biomarkers, and Treatments. Int. Immunopharmacology 84, 106535. 10.1016/j.intimp.2020.106535 32361569

[B7] Bianchini G. Balko J. M. Mayer I. A. Sanders M. E. Gianni L. (2016). Triple-negative Breast Cancer: Challenges and Opportunities of a Heterogeneous Disease. Nat. Rev. Clin. Oncol. 13 (11), 674–690. 10.1038/nrclinonc.2016.66 27184417 PMC5461122

[B8] Chang G. Shi L. Ye Y. Shi H. Zeng L. Tiwary S. (2020). YTHDF3 Induces the Translation of m6A-Enriched Gene Transcripts to Promote Breast Cancer Brain Metastasis. Cancer Cell 38 (6), 857–871. 10.1016/j.ccell.2020.10.004 33125861 PMC7738369

[B9] Chen Y. Lin Y. Shu Y. He J. Gao W. (2020). Interaction between N6-Methyladenosine (m6A) Modification and Noncoding RNAs in Cancer. Mol. Cancer 19 (1), 94. 10.1186/s12943-020-01207-4 32443966 PMC7243333

[B10] Coker H. Wei G. Brockdorff N. (2019). m6A Modification of Non-coding RNA and the Control of Mammalian Gene Expression. Biochim. Biophys. Acta (Bba) - Gene Regul. Mech. 1862 (3), 310–318. 10.1016/j.bbagrm.2018.12.002 30550772

[B11] Coots R. A. Liu X.-M. Mao Y. Dong L. Zhou J. Wan J. (2017). m6A Facilitates eIF4F-independent mRNA Translation. Mol. Cel 68 (3), 504–514. e507. 10.1016/j.molcel.2017.10.002 PMC5913006 29107534

[B12] Desrosiers R. Friderici K. Rottman F. (1974). Identification of Methylated Nucleosides in Messenger RNA from Novikoff Hepatoma Cells. Proc. Natl. Acad. Sci. U.S.A. 71 (10), 3971–3975. 10.1073/pnas.71.10.3971 4372599 PMC434308

[B13] Emens L. A. Cruz C. Eder J. P. Braiteh F. Chung C. Tolaney S. M. (2019). Long-term Clinical Outcomes and Biomarker Analyses of Atezolizumab Therapy for Patients with Metastatic Triple-Negative Breast Cancer. JAMA Oncol. 5 (1), 74–82. 10.1001/jamaoncol.2018.4224 30242306 PMC6439773

[B14] Geeleher P. Cox N. J. Huang R. (2014). Clinical Drug Response Can Be Predicted Using Baseline Gene Expression Levels and *In Vitro* Drug Sensitivity in Cell Lines. Genome Biol. 15 (3), R47. 10.1186/gb-2014-15-3-r47 24580837 PMC4054092

[B15] Hanna A. Balko J. M. (2021). Breast Cancer Resistance Mechanisms: Challenges to Immunotherapy. Breast Cancer Res. Treat. 190, 5–17. 10.1007/s10549-021-06337-x 34322780 PMC8560575

[B16] Harbeck N. Penault-Llorca F. Cortes J. Gnant M. Houssami N. Poortmans P. (2019). Breast Cancer. Nat. Rev. Dis. Primers 5 (1), 66. 10.1038/s41572-019-0111-2 31548545

[B17] He X. Tan L. Ni J. Shen G. (2021). Expression Pattern of m6A Regulators Is Significantly Correlated with Malignancy and Antitumor Immune Response of Breast Cancer. Cancer Gene Ther. 28 (3-4), 188–196. 10.1038/s41417-020-00208-1 32759989 PMC8057950

[B18] Huang H. Weng H. Chen J. (2020). m6A Modification in Coding and Non-coding RNAs: Roles and Therapeutic Implications in Cancer. Cancer Cell 37 (3), 270–288. 10.1016/j.ccell.2020.02.004 32183948 PMC7141420

[B19] Klinge C. M. Piell K. M. Tooley C. S. Rouchka E. C. (2019). HNRNPA2/B1 Is Upregulated in Endocrine-Resistant LCC9 Breast Cancer Cells and Alters the miRNA Transcriptome when Overexpressed in MCF-7 Cells. Sci. Rep. 9 (1), 9430. 10.1038/s41598-019-45636-8 31263129 PMC6603045

[B20] Lainetti P. d. F. Leis-Filho A. F. Laufer-Amorim R. Battazza A. Fonseca-Alves C. E. (2020). Mechanisms of Resistance to Chemotherapy in Breast Cancer and Possible Targets in Drug Delivery Systems. Pharmaceutics 12 (12), 1193. 10.3390/pharmaceutics12121193 PMC7763855 33316872

[B21] Lehmann B. D. Bauer J. A. Chen X. Sanders M. E. Chakravarthy A. B. Shyr Y. (2011). Identification of Human Triple-Negative Breast Cancer Subtypes and Preclinical Models for Selection of Targeted Therapies. J. Clin. Invest. 121 (7), 2750–2767. 10.1172/JCI45014 21633166 PMC3127435

[B22] Li A. Chen Y.-S. Ping X.-L. Yang X. Xiao W. Yang Y. (2017). Cytoplasmic m6A Reader YTHDF3 Promotes mRNA Translation. Cell Res 27 (3), 444–447. 10.1038/cr.2017.10 28106076 PMC5339832

[B23] Li Y. Xiao J. Bai J. Tian Y. Qu Y. Chen X. (2019). Molecular Characterization and Clinical Relevance of m6A Regulators across 33 Cancer Types. Mol. Cancer 18 (1), 137. 10.1186/s12943-019-1066-3 31521193 PMC6744659

[B24] Liu N. Dai Q. Zheng G. He C. Parisien M. Pan T. (2015). N6-methyladenosine-dependent RNA Structural Switches Regulate RNA-Protein Interactions. Nature 518 (7540), 560–564. 10.1038/nature14234 25719671 PMC4355918

[B25] Liu N. Zhou K. I. Parisien M. Dai Q. Diatchenko L. Pan T. (2017). N 6-methyladenosine Alters RNA Structure to Regulate Binding of a Low-Complexity Protein. Nucleic Acids Res. 45 (10), 6051–6063. 10.1093/nar/gkx141 28334903 PMC5449601

[B26] Loibl S. Gianni L. (2017). HER2-positive Breast Cancer. The Lancet 389 (10087), 2415–2429. 10.1016/s0140-6736(16)32417-5 27939064

[B27] Metzger-Filho O. Tutt A. de Azambuja E. Saini K. S. Viale G. Loi S. (2012). Dissecting the Heterogeneity of Triple-Negative Breast Cancer. Jco 30 (15), 1879–1887. 10.1200/JCO.2011.38.2010 22454417

[B28] Pan Y. Ma P. Liu Y. Li W. Shu Y. (2018). Multiple Functions of m6A RNA Methylation in Cancer. J. Hematol. Oncol. 11 (1), 48. 10.1186/s13045-018-0590-8 29587823 PMC5870302

[B29] Perou C. M. Sørlie T. Eisen M. B. van de Rijn M. Jeffrey S. S. Rees C. A. (2000). Molecular Portraits of Human Breast Tumours. Nature 406 (6797), 747–752. 10.1038/35021093 10963602

[B30] Prabhu J. S. Korlimarla A. Anupama C. E. Alexander A. Raghavan R. Kaul R. (2017). Dissecting the Biological Heterogeneity within Hormone Receptor Positive HER2 Negative Breast Cancer by Gene Expression Markers Identifies Indolent Tumors within Late Stage Disease. Translational Oncol. 10 (4), 699–706. 10.1016/j.tranon.2017.04.011 PMC5506875 28704710

[B31] Russnes H. G. Navin N. Hicks J. Borresen-Dale A.-L. (2011). Insight into the Heterogeneity of Breast Cancer through Next-Generation Sequencing. J. Clin. Invest. 121 (10), 3810–3818. 10.1172/JCI57088 21965338 PMC3195464

[B32] Shi H. Wang X. Lu Z. Zhao B. S. Ma H. Hsu P. J. (2017). YTHDF3 Facilitates Translation and Decay of N^6^-Methyladenosine-Modified RNA. Cel Res 27 (3), 315–328. 10.1038/cr.2017.15 PMC5339834 28106072

[B33] Sørlie T. Perou C. M. Tibshirani R. Aas T. Geisler S. Johnsen H. (2001). Gene Expression Patterns of Breast Carcinomas Distinguish Tumor Subclasses with Clinical Implications. Proc. Natl. Acad. Sci. U.S.A. 98 (19), 10869–10874. 10.1073/pnas.191367098 11553815 PMC58566

[B34] Stanton S. E. Disis M. L. (2016). Clinical Significance of Tumor-Infiltrating Lymphocytes in Breast Cancer. J. Immunotherapy Cancer 4, 59. 10.1186/s40425-016-0165-6 PMC5067916 27777769

[B35] Sung H. Ferlay J. Siegel R. L. Laversanne M. Soerjomataram I. Jemal A. (2021). Global Cancer Statistics 2020: GLOBOCAN Estimates of Incidence and Mortality Worldwide for 36 Cancers in 185 Countries. CA A. Cancer J. Clin. 71, 209–249. 10.3322/caac.21660 33538338

[B36] Wei C.-M. Gershowitz A. Moss B. (1975). Methylated Nucleotides Block 5′ Terminus of HeLa Cell Messenger RNA. Cell 4 (4), 379–386. 10.1016/0092-8674(75)90158-0 164293

[B37] Wu R. Jiang D. Wang Y. Wang X. (2016). N 6-Methyladenosine (m6A) Methylation in mRNA with A Dynamic and Reversible Epigenetic Modification. Mol. Biotechnol. 58 (7), 450–459. 10.1007/s12033-016-9947-9 27179969

[B38] Xu X. De Angelis C. Burke K. A. Nardone A. Hu H. Qin L. (2017). HER2 Reactivation through Acquisition of the HER2 L755S Mutation as a Mechanism of Acquired Resistance to HER2-Targeted Therapy in HER2^+^ Breast Cancer. Clin. Cancer Res. 23 (17), 5123–5134. 10.1158/1078-0432.CCR-16-2191 28487443 PMC5762201

[B39] Yang Y. Fan X. Mao M. Song X. Wu P. Zhang Y. (2017). Extensive Translation of Circular RNAs Driven by N^6^-Methyladenosine. Cel Res 27 (5), 626–641. 10.1038/cr.2017.31 PMC5520850 28281539

[B40] Zhao N. Rosen J. M. (2021). Breast Cancer Heterogeneity through the Lens of Single-Cell Analysis and Spatial Pathologies. Semin. Cancer Biol. 10.1016/j.semcancer.2021.07.010 PMC8761219 34274486

[B41] Zhu Y. Zhu X. Tang C. Guan X. Zhang W. (2021). Progress and Challenges of Immunotherapy in Triple-Negative Breast Cancer. Biochim. Biophys. Acta (Bba) - Rev. Cancer 1876 (2), 188593. 10.1016/j.bbcan.2021.188593 34280474

